# BCAT1 Associates with DNA Repair Proteins KU70 and KU80 and Contributes to Regulate DNA Repair in T-Cell Acute Lymphoblastic Leukemia (T-ALL)

**DOI:** 10.3390/ijms252413571

**Published:** 2024-12-18

**Authors:** Valeria Tosello, Chiara Rompietti, Adonia E. Papathanassiu, Giorgio Arrigoni, Erich Piovan

**Affiliations:** 1 Basic and Translational Oncology Unit, Veneto Institute of Oncology IOV—IRCCS, 35127 Padua, Italy; valeria.tosello@iov.veneto.it; 2 Immunology and Molecular Oncology Diagnostics, Veneto Institute of Oncology IOV—IRCCS, 35128 Padua, Italy; chiara.rompietti@iov.veneto.it; 3 Ergon Pharmaceuticals, LLC., Washington, DC 20011, USA; adoniap@ergonpharma.com; 4 Department of Biomedical Sciences, University of Padua, 35122 Padua, Italy; giorgio.arrigoni@unipd.it; 5 Proteomics Center, University of Padua and Azienda Ospedaliera of Padua, 35131 Padua, Italy; 6 Department of Surgery, Oncology and Gastroenterology, University of Padua, 35128 Padua, Italy

**Keywords:** T-cell lymphoblastic leukemia, metabolism, BCAT1, leukemia growth

## Abstract

Increased expression of branched-chain amino acid (BCAA) transaminase 1 (BCAT1) often correlates with tumor aggressiveness and drug resistance in cancer. We have recently reported that BCAT1 was overexpressed in a subgroup of T-cell acute lymphoblastic (T-ALL) samples, especially those with NOTCH1 activating mutations. Interestingly, BCAT1-depleted cells showed pronounced sensitivity to DNA-damaging agents such as etoposide; however, how BCAT1 regulates this sensitivity remains uncertain. Here, we provide further clues on its chemo-sensitizing effect. Indeed, BCAT1 protein regulates the non-homologous end joining (c-NHEJ) DNA repair pathway by physically associating with the KU70/KU80 heterodimer. BCAT1 inhibition during active repair of DNA double-strand breaks (DSBs) led to increased KU70/KU80 acetylation and impaired c-NHEJ repair, a dramatic increase in DSBs, and ultimately cell death. Our results suggest that, in T-ALL, BCAT1 possesses non-metabolic functions that confer a drug resistance mechanism and that targeting BCAT1 activity presents a novel strategy to improve chemotherapy response in T-ALL patients.

## 1. Introduction

T-cell acute lymphoblastic leukemia (T-ALL) is an aggressive hematological malignancy accounting for ≈15% of pediatric and ≈25% of adult ALL cases, treated with intensive chemotherapy regimens [1,2]. Although advances in therapeutic regimens have been recorded, a significant fraction of patients relapse and/or present life-threatening therapy-related toxicities. Genetically, T-ALL is a heterogeneous disease, where a wide spectrum of genetic alterations and microenvironmental cues cooperate to induce leukemia [3,4]. Gain-of-function mutations in *NOTCH1* are highly prevalent genetic lesions found in T-ALL [5]. *NOTCH1* mutations hijack the physiological role of NOTCH signaling during thymic development in promoting T-cell lineage specification, cell growth, and proliferation. Constitutive NOTCH1 signaling in T-ALL determines the transcriptional activation of an array of anabolic pathways involved in cell growth, including ribosome biosynthesis, protein translation, and nucleotide and amino acid metabolism [6,7]. Interestingly, NOTCH1 is a negative regulator of DNA damage response (DDR) through binding and inhibiting of the ATM (Ataxia-Telangiectasia Mutated) Ser/Thr kinase [8]. This supports a role for NOTCH1 activation in favoring the onset of genetic alterations in T-ALL, which are thought to be driven by errant DNA repair.

Branched-chain amino acid transaminase 1 (BCAT1) is the cytosolic enzyme commonly responsible for the reversible transfer of an amino group from leucine, isoleucine, and valine to alpha-ketoglutarate (α-KG) to form glutamate and the corresponding α-ketoacid [9]. We recently reported that Bcat1 was overexpressed following NOTCH1-induced transformation of leukemic progenitors and that NOTCH1 directly controlled BCAT1 expression by binding to a *BCAT1* promoter [10]. In murine T-ALL cells, Bcat1 depletion or inhibition redirected leucine metabolism towards production of 3-hydroxy butyrate (3-HB). Further, BCAT1-depleted cells showed altered protein acetylation levels, which correlated with an increased sensitivity to DNA-damaging agents such as etoposide. Here, we provide further insight on how BCAT1 regulates chemosensitivity in T-ALL cells. Specifically, we found that BCAT1 depletion was associated with ineffective DNA damage signaling and transcriptional response following DNA damage. BCAT1 was found to physically associate with the XRCC5 (KU80) and XRCC6 (KU70) proteins implicated in non-homologous end joining (c-NHEJ). BCAT1 depletion led to increased KU70 acetylation, impaired c-NHEJ repair with increased DNA damage and cell death, especially when combined with a genotoxic insult.

## 2. Results

### 2.1. BCAT1 Depletion Activates an Ineffective DNA Damage Transcriptional Response

**Figure 1:**
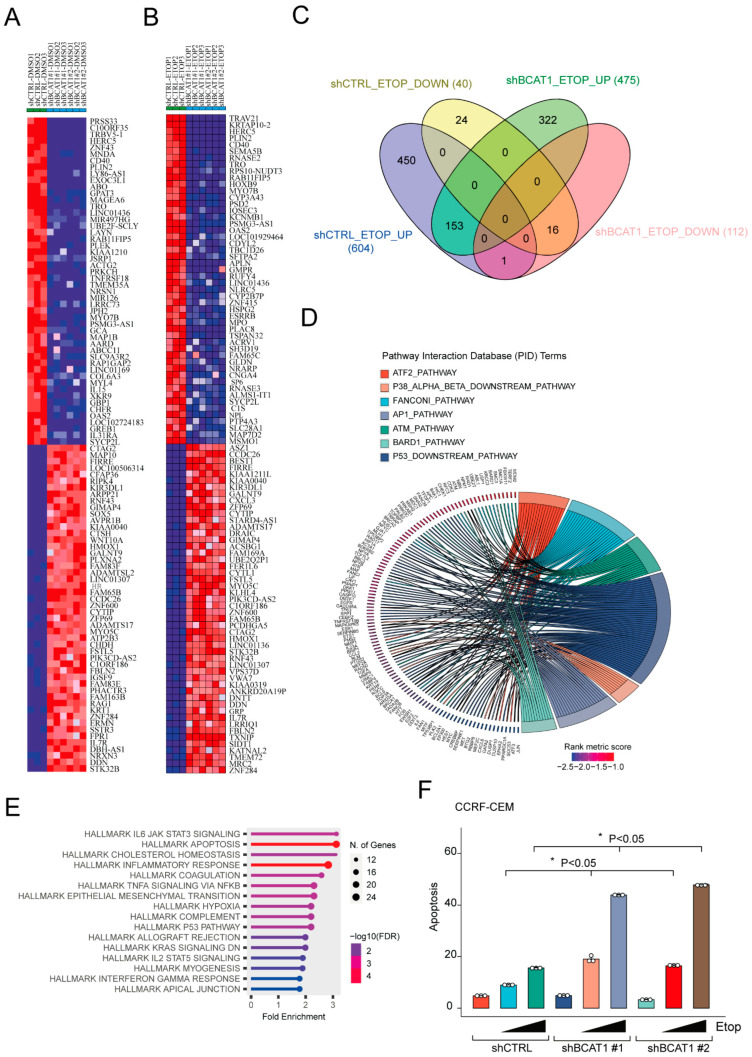
*BCAT1* loss induces a dysfunctional DNA damage response following etoposide treatment. (**A**) Heat map representation of the top down- and upregulated genes between control CCRF-CEM (shCTRL) and BCAT1-depleted CCRF-CEM cells (sh*BCAT1* #1 and #2). (**B**) Heat map representation of the top down– and upregulated genes between CCRF–CEM shCTRL treated with etoposide for 24 h and CCRF-CEM sh*BCAT1* treated with etoposide for 24 h. (**C**) Venn diagram comparing number of transcripts significantly up- (UP) or downregulated (DOWN) by etoposide treatment in each dataset. (**D**,**E**) ShinyGO 0.8 software was used for enrichment analysis of differentially expressed genes between control and BCAT1−depleted cells treated with etoposide. Gene set enrichment analysis (GSEA) identified significantly enriched Pathway Interaction Database (PID) and hallmark gene sets. (**D**) The chord plot shows the relationship between PID gene sets and genes. (**E**) Lollipop plots showing enriched hallmark gene sets. (**F**) Quantification of apoptosis in CCRF-CEM T-ALL cells transduced with shCTRL or sh*BCAT1* and treated in vitro for 48 h with DMSO (vehicle) or etoposide (500 nM–1 μM). Significance was calculated using the Kruskal-Wallis test. * *p* < 0.05.

We recently described that BCAT1 depletion sensitizes T-ALL cells to DNA-damaging agents such as etoposide [10]. The mechanism behind this effect is still ill-defined. To gain further insight on the cellular mechanisms involved in BCAT1-mediated chemoresistance, we performed gene expression profiling by mRNA-seq in a T-ALL cell line expressing high levels of BCAT1 and relatively resistant to DNA-damaging agents such as CCRF-CEM cells. These cells were transduced with control or two *BCAT1*-targeting shRNAs and treated with etoposide or vehicle for 24 h. Comparison of gene expression profiles showed that *BCAT1* knockdown or etoposide treatment determined significant transcriptome-wide alterations (Figure 1A,B, and Supplementary Tables S1 and S2; ≥2-fold change, *p* < 0.05, FDR < 0.05). When comparing the overlap of modulated transcripts between etoposide-treated control and BCAT1-depleted cells, we found 153 transcripts commonly upregulated in both groups, while 322 and 450 transcripts were uniquely upregulated in BCAT1-depleted and control cells, respectively (Figure 1C). Gene set enrichment analysis (GSEA) of differentially expressed genes between control and BCAT1-depleted cells treated with etoposide disclosed that numerous gene sets related to DNA damage/repair signaling, including the “ATM pathway”, “BARD1 pathway”, and “p53 downstream pathway” were enriched in *BCAT1* shRNA cells (Figure 1D). Interestingly, the “Apoptosis signaling” and “p53 signaling” pathways were amongst the top hallmark gene sets enriched in sh*BCAT1* cells (Figure 1E). Thus, differential gene expression analysis seems to suggest an enhanced but ineffective DNA damage response (DDR) leading to cell death in BCAT1-depleted T-ALL cells (Figure 1F and Supplementary Figure S1A). This leads to increased drug sensitivity (lower IC_50_, Supplementary Figure S1B).

### 2.2. BCAT1 Binds with the KU Complex and Modulates DNA Damage Repair Signaling Pathways

**Figure 2:**
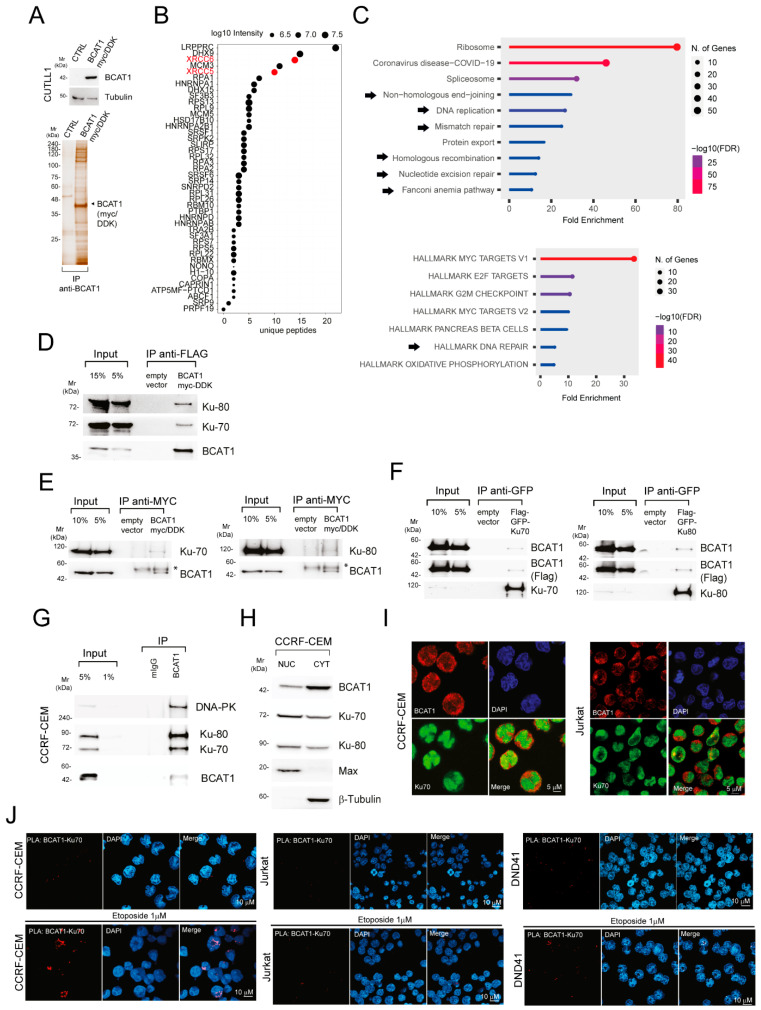
BCAT1 interacts with KU70 and KU80 proteins. (**A**) Purification of BCAT1 interacting partners. Expression of BCAT1 and tubulin was analyzed by immunoblotting (top) in CUTLL1 T-ALL cells stably expressing empty vector or double epitope-tagged BCAT1 (myc/DDK). Cytoplasmic extracts prepared from BCAT1 myc/DDK-expressing or mock-transduced (CTRL) CUTLL1 cells were subjected to sequential immunoprecipitation (IP) using anti-FLAG and anti-MYC beads. Proteins were resolved by SDS-PAGE and visualized by silver staining (bottom). Molecular weights (Mr) are indicated on the left. (**B**) Top proteins interacting with BCAT1 identified by mass spectrometry based on the number of unique peptides and intensity (bubble plot). These proteins were not identified in the control IP. Results are from one experiment of two performed with similar results. (**C**) ShinyGO 0.8 software was used for enrichment analysis of BCAT1-interacting partners. KEGG pathway (top) and hallmark (bottom) analysis representing top significantly enriched pathways (lollipop plots). (**D**) Cytoplasmic extracts prepared from CUTLL1 T-ALL cells stably expressing empty vector or BCAT1 myc/DDK were subjected to IP using anti-FLAG beads. FLAG peptide was used to elute proteins from the beads and were resolved by SDS-PAGE. Immunoblotting for BCAT1, KU70, and KU80 proteins was performed. (**E**) HEK 293T cells stably expressing epitope-tagged BCAT1 were transfected with GFP-KU70 (left) or GFP-KU80 (right) expression vectors, and lysates were subjected to co-immunoprecipitation (Co-IP) using anti-MYC tag beads. Immunoblot analysis for BCAT1 (anti-BCAT1) and KU70 or KU80 (anti-GFP) was performed. Asterisk (*) indicates non-specific bands. (**F**) HEK 293T cells stably expressing epitope-tagged BCAT1 were transfected with GFP-KU70 (left) or GFP-KU80 (right) expression vectors, and lysates were subjected to Co-IP using anti-GFP beads. Immunoblot analysis for BCAT1 (anti-BCAT1 and anti-Flag) and KU70 or KU80 (anti-GFP) was performed. (**G**) Interaction between endogenously expressed BCAT1, KU70, and KU80 proteins in CCRF-CEM cells was demonstrated by IP with IgG (mIgG) and anti-BCAT1 antibody followed by immunoblot analysis with the indicated antibodies. DNA-PKcs was also detected. (**H**) Cellular localization analysis of BCAT1, KU70, and KU80 via Western blot analysis of nuclear and cytoplasmic cell fractions in cell lysates from CCRF-CEM T-ALL cells. Tubulin and Max proteins are shown as controls for cytosolic and nuclear fractions. CYT: cytoplasmic fraction; NUC: nuclear fraction. (**I**) Localization of BCAT1 (red) and KU70 (green) by immunofluorescence in CCRF-CEM and Jurkat T-ALL cells. DAPI (blue) was used as a nuclear marker. A scale bar is shown. (**J**) The interaction between BCAT1 and KU70 was assessed under basal conditions (top) and after 24 h of treatment with etoposide (1 µM; bottom) by proximity ligation assay (PLA) with the indicated pairs of primary antibodies. DAPI (blue) was used as a nuclear marker. Scale bar is shown.

To try and address how BCAT1 may regulate the DDR, we reasoned that BCAT1 may do this through a “moonlighting”/non-canonical function, possibly by complexing with distinct protein partners [11,12]. We thus explored the BCAT1 interactome by immunoprecipitation (IP) using tandem affinity purification (TAP) of double-tagged BCAT1 (BCAT1 myc-DDK) (Figure 2A). This was performed in CUTLL1 T-ALL cells that express undetectable levels of endogenous BCAT1 (Figure 2A). Cytoplasmic extracts were subjected to TAP and LC-MS/MS analysis. We identified 129 candidate interacting proteins (≥10 enrichment compared to control IP), of which n = 44 were undetectable in the control IP (Supplementary Table S3 and Figure 2B) and found the KU70 (XRCC6) and KU80 (XRCC5) proteins to be highly prevalent (Figure 2B). Interrogating the Kyoto Encyclopedia of Genes and Genomes (KEGG) or the hallmark SigDB databases using ShinyGO 0.80 [13] revealed several enriched pathways associated with the BCAT1 interactome, with the majority of them being linked to DNA repair mechanisms (Figure 2C). These results are compatible with our previous results (see above) and the recently described phenotype of BCAT1-deficient T-ALL cells [10], where a defective DNA repair results in apoptosis [14]. 

Given the essential role of DNA repair pathways in tumor initiation and progression, we focused our investigation on the functional consequence of BCAT1-KU complex interaction. First, we validated the interaction of BCAT1 with the KU70 and KU80 proteins using co-immunoprecipitation (Co-IP) in two human cell lines, the T-cell lymphoma CUTTL1 and embryonic kidney HEK 293T, expressing tagged BCAT1 (Figure 2D,E). Likewise, ectopically expressed GFP/FLAG-KU70 or KU80 proteins co-immunoprecipitated with BCAT1 in HEK 293T (Figure 2F). A comparable interaction was observed in the T-ALL cell line CCRF-CEM (Figure 2G). Although KU70 and KU80 subunits are predominantly localized in the nucleus, they are also present in the cytoplasm and at the cytoplasmic membrane, where they have been implicated in numerous cellular processes [15]. On the other hand, while BCAT1 is predominantly cytosolic, some protein pools have been localized in the cytoplasmic membrane and the nucleus [16]. We thus evaluated the subcellular localization of KU70/KU80 and BCAT1 in T-ALL cells. As reported in other cell types, a fraction of BCAT1 was nuclear and a fraction of KU70/KU80 proteins was cytoplasmic in T-ALL cell lines and patient derived xenografts (PDXs) (Figure 2H,I and Supplementary Figure S2A,B). These results render an interaction between these proteins feasible. Proximity ligation assays (PLA) corroborated the presence of a weak interaction between KU70 and BCAT1 proteins in the cytoplasm of T-ALL cells under basal conditions, which became more pronounced and nuclear following DNA damage (Figure 2J). 

**Figure 3:**
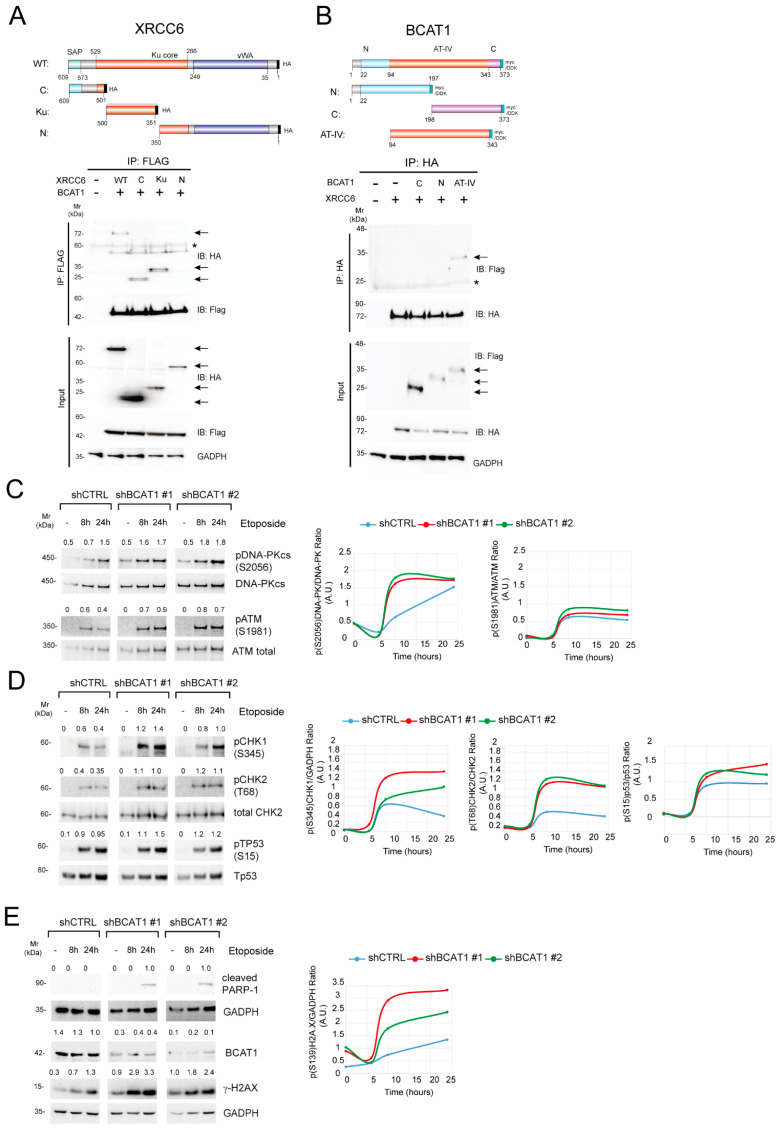
BCAT1–depletion induces a dysfunctional DNA damage response following etoposide treatment. (**A**) Schematic representations of the plasmids encoding full-length (WT) and truncation mutants of XRCC6 (top). vWA: von Willebrand A domain; SAP: SAF-A/B, Acinus, and PIAS domain. HEK 293T cells stably expressing epitope-tagged BCAT1 were transfected with the indicated plasmid. Cell lysates were subjected to IP with anti-FLAG beads followed by immunoblot analysis with the indicated antibodies. The arrows indicate expected positions of the respective proteins, and asterisks (*) indicate non–specific bands. (**B**) Schematic representations of the plasmids encoding full-length (WT) and truncation mutants of BCAT1 (top). N: Branched–chain amino acid aminotransferase-like N-terminal domain; AT–IV: aminotransferase class IV domain; C: Branched-chain amino acid aminotransferase-like C–terminal domain. HEK 293T cells were transfected with HA–tagged XRCC6 and the indicated BCAT1 mutant plasmids. Cell lysates were subjected to IP with anti-HA beads followed by immunoblot analysis with the indicated antibodies. The arrows indicate expected positions of the respective proteins, and asterisks (*) indicate non-specific bands. (**C**–**E**) CCRF–CEM T-ALL cells transduced with shCTRL or sh*BCAT1* were treated with 1 µM etoposide for the indicated time. Subsequently, whole cell lysates were collected and analyzed by immunoblotting for proteins implicated in (**C**,**D**) the activation of the DNA damage response (pDNA-PKcs, pATM, pCHK1, pCHK2, pTP53); (**E**) DNA damage (γH2AX) and apoptosis (cleaved PARP-1). Total DNA–PKcs and ATM are shown as loading controls (**C**). Total CHK2, total TP53, and GADPH are shown as loading controls (**D**,**E**). Phospho-protein/protein ratios are shown (top) in each panel. A graphical representation of the phospho-protein/protein ratios is also shown for selected proteins (right panels).

To gain further insight on the KU70/KU80-BCAT1 interaction, truncation mutants of KU70 and KU80 were generated to determine which region was required for the interaction. Anti-FLAG IP from the lysates of cells, transiently transfected with HA-tagged KU70 full length or mutants (aa 1–350; aa 351–500; aa 501–609), followed by immunoblotting with anti-HA antibodies, disclosed that Ku and C-terminal domains (aa 351–609) of KU70 interact with BCAT1 (Figure 3A). The BCAT1-binding region of KU80 was similarly determined using HA-tagged KU80 full-length protein and deletion mutants (aa 1–240; aa 241–550; aa 551–732). In that instance, it was the N-terminal domain (aa 1–240) of KU80 that interacted with BCAT1 (Supplementary Figure S3A). We also mapped the region of BCAT1 that interacts with the KU70/KU80 proteins. Expression constructs encoding myc-DDK-tagged N-terminal BCAT1 (aa 1–197), C-terminal (aa 198–373), and aminotransferase IV (AT IV) domain (aa 94–343) were generated and used for IP with an anti-HA antibody in 293T cells. Interestingly, only the aminotransferase IV domain BCAT1 mutant showed binding ability to both KU70 and KU80 proteins (Figure 3B, Supplementary Figure S3B). These results also suggest that the amino acids neighboring the central portion of BCAT1 (aa. 197–198) are necessary for these interactions. The ability of BCAT1 to complex with KU70 and KU80 suggests that the former directly regulates the c-NHEJ repair pathway. This is an interesting role for a primarily cytosolic enzyme to play on a typically nuclear process.

Further, BCAT1 depletion seems to promote an ineffective DDR following etoposide treatment. To document this phenomenon better, we determined downstream DDR signaling following etoposide treatment. We thus examined the phosphorylation status of key proteins that control activation of different DNA DSB repair pathways (DNA-PK for the c-NHEJ pathway and ATM for the homologous recombination (HR) pathway) or cell cycle arrest (CHK1, CHK2, and p53). Figure 3C indicates that both the c-NHEJ and the HR pathways were involved in repairing etoposide-induced DNA damage. Whereas control and BCAT1-depleted CCRF-CEM cells exhibited similar rates of ATM phosphorylation, DNA-PK was subject to an accelerated phosphorylation that reached a similar maximum as the control cells but at an earlier time point (8 h vs. 24 h; Figure 3C). The combination of *BCAT1* silencing and etoposide treatment was also associated with increased levels of phosphorylated CHK1 and CHK2, while the effects on p53 phosphorylation were less evident (Figure 3D). Interestingly, accelerated c-NHEJ signaling was not associated with DNA DSB repair but rather with further DNA damage, as suggested by the dramatic increase in the levels of γH2AX in BCAT1-depleted cells compared to control cells (Figure 3E). Cleaved PARP-1 was observed only at 24 h of etoposide treatment, indicating that in BCAT1-depleted cells, the failure to repair DNA DSBs leads to extensive DNA damage and eventually apoptosis (Figure 3E). 

### 2.3. BCAT1 Depletion in Human T-ALL Leads to Reduced c-NHEJ Repair with Increased KU70/KU80 Acetylation

**Figure 4:**
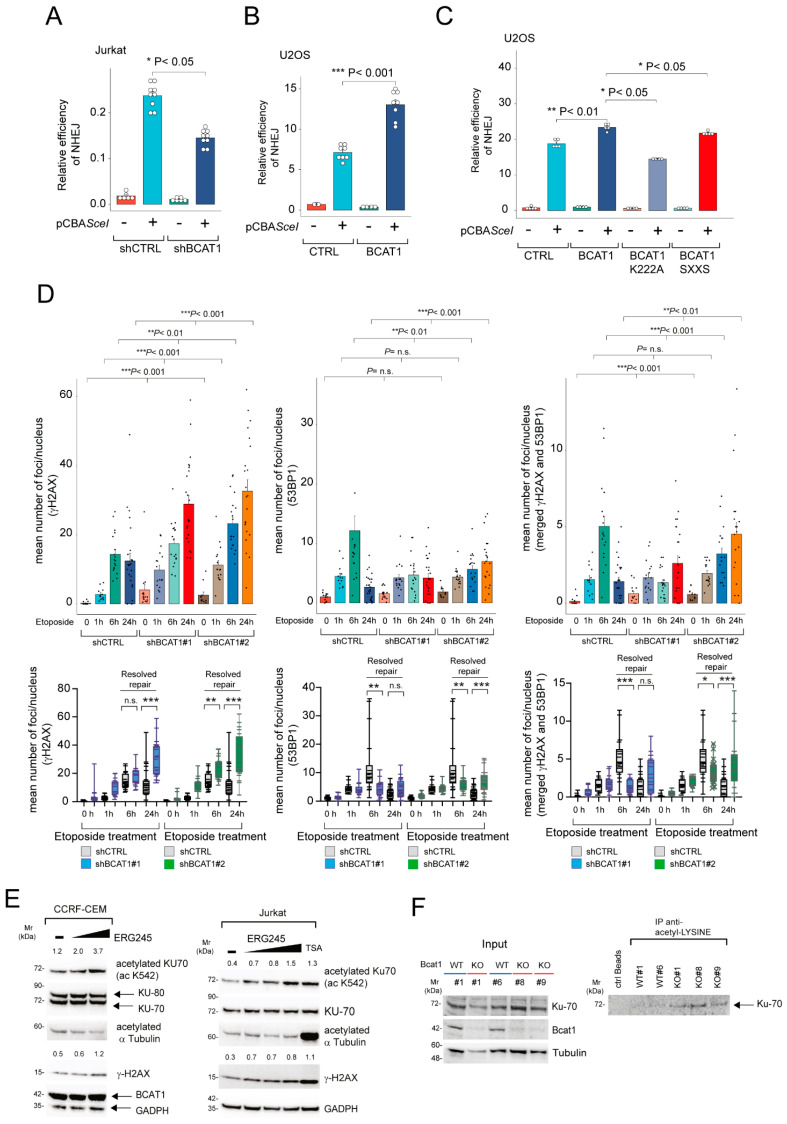
BCAT1-depletion decreases DNA repair by modulating KU70 acetylation levels. (**A**) Jurkat reporter cell lines were generated from parental cell lines by transfection of the pimEJ5-GFP construct and subsequent selection with puromycin for over 14 days. These cell lines were subsequently engineered to lose BCAT1 expression (pLKO.1 sh*BCAT1*#3). The reporter cell lines were then electroporated with the pCBA-SceI endonuclease-expressing vector (or empty vector). After 72 h, the activity of the c-NHEJ (pimEJ5-GFP vector-expressing cells) DNA repair pathway was assessed by measuring the percentage of GFP-positive cells using flow cytometry. Error bars indicate ±SD. Results from one of three independent experiments performed with 6–9 replicates are shown. Significance was calculated using an unpaired Mann–Whitney *U* test. * *p* < 0.05. (**B**) U2OS cells were engineered to overexpress BCAT1 (BCAT1 myc/DDK). Cells were then transfected with the pimEJ5-GFP vector and pCBA-SceI or empty vector. After 48 h, the activity of the c-NHEJ (pimEJ5–GFP vector-expressing cells) DNA repair pathway was assessed by measuring the percentage of GFP-positive cells using flow cytometry. Error bars indicate ± SD. Results from one of two independent experiments are shown. Significance was calculated using an unpaired Mann-Whitney *U* test. *** *p* < 0.001. (**C**) U2OS cells were engineered to overexpress BCAT1 (BCAT1 myc/DDK) or BCAT1 mutants (K222A, SXXS). Cells were then transfected with the pimEJ5-GFP vector and pCBA-SceI or empty vector. After 48 h, the activity of the c-NHEJ (pimEJ5–GFP vector-expressing cells) DNA repair pathway was assessed by measuring the percentage of GFP–positive cells using flow cytometry. Error bars indicate ± SD. Results from one of two independent experiments are shown. Significance was calculated using an unpaired Mann-Whitney *U* test. * *p* < 0.05, ** *p* < 0.01. (**D**) Kinetics of DNA repair in CCRF-CEM control and *BCAT1* stable knockdown T-ALL cells (sh*BCAT1*#1, sh*BCAT1*#2). The number of γH2AX foci (left), 53BP1 foci (middle), and coincident γH2AX/53BP1 foci (right) per nucleus following etoposide treatment are denoted. Each point represents data from a single cell, and the bars denote the median foci number per cell. Top panels: Significance was calculated using the Kruskal-Wallis test. ** *p* < 0.01, *** *p* < 0.001. n.s. = not significant. Box–and–whisker plots denote expression from minimum to maximum (bottom). Significance was calculated using an unpaired Mann-Whitney *U* test. ** *p* < 0.01, *** *p* < 0.001. n.s. = not significant. (**E**) CCRF-CEM T-ALL cells (left) were treated with different doses of ERG245 (100–200 µM) for 24 h. Subsequently, whole cell lysates were collected and analyzed by immunoblotting for the indicated proteins. Total KU70 and GADPH are shown as loading controls. Jurkat T-ALL cells (right) were treated with different doses of ERG245 (100–300 µM) or Trichostatin A (TSA; 100 nM) for 24 h. Subsequently, whole cell lysates were collected and analyzed by immunoblotting for the indicated proteins. Total KU70 and GADPH are shown as loading controls. The acetylated KU70/total KU70 protein ratios and γH2AX/GADPH protein ratios are also shown. (**F**) Whole cell lysates from ΔE-NOTCH1 leukemias wild-type and KO for *Bcat1* were immunoprecipitated using anti-acetyl-lysine affinity beads or control beads and probed for Ku70 and Bcat1. α-Tubulin is shown as a loading control (input).

Binding of KU70/KU80 to DNA break ends is required to initiate c-NHEJ-mediated DSB repair [17] and prevents additional DSB end resection that facilitates HR. The data above suggest that BCAT1 may alter the activity of DSB repair pathways, especially c-NHEJ. Stable *BCAT1* knockdown or shRNA-expressing control cells co-expressing a plasmid-based GFP reporter specific for assessing c-NHEJ-mediated DSB repair (pimEJ5-GFP, which contains two recognition sites for I-SceI endonuclease) [18,19] were generated. Using these cells, we were able to evaluate the efficiency of c-NHEJ repair based on the number of GFP-positive cells measured by flow cytometry. Plasmid-based repair assays showed that Jurkat T-ALL cells exhibited reduced c-NHEJ repair following stable *BCAT1* knockdown (Figure 4A). Similarly, plasmid-based repair assays in BCAT1-overexpressing U2OS cells (that present low protein levels of endogenous BCAT1) demonstrated increased c-NHEJ repair activity (Figure 4B). This activity was highly dependent on the enzymatic activity of BCAT1 and less on its peroxide-sensitive, redox-active CXXC motif [11,16], as the K222A mutant completely abrogated the increase in c-NHEJ repair activity (Figure 4C).

To independently confirm a role for BCAT1 in modulating DNA DSB repair, we assessed the kinetics and localization of 53BP1 and γH2AX in etoposide-treated CCRF-CEM T-ALL cells following stable knockdown of *BCAT1*. 53BP1 and γH2AX are recruited to DNA following induction of DSBs, and their kinetics can be used to quantify resolution of the breaks following exposure to DNA-damaging agents [20,21]. Using this strategy, we found that BCAT1 depletion led to a significantly slower DNA break resolution (Figure 4D). The overall numbers of γH2AX foci were increased at most early time points and reached a maximum at the latest time point (24 h) in BCAT1-depleted cells (Figure 4D). A similar pattern was observed for 53BP1 foci (and co-localized 53BP1/γH2AX foci), which were persistently elevated also at the latest time point (24 h). 

This probably reflects impaired/slower repair in BCAT1-depleted cells following etoposide-induced DSB damage. Together, our data suggest that BCAT1 positively regulates the c-NHEJ pathway activity in human T-ALL and that BCAT1-depleted human T-ALLs are locked in a state of dampened DSB DNA repair.

KU70 acetylation has been shown to impair c-NHEJ activity by modifying the KU70 lysine residues needed for binding dsDNA ends [22,23]. Recently, we reported [10] that 3-HB (a HDAC inhibitor [24]) accumulates following Bcat1 inhibition in murine T-ALL. We thus evaluated the acetylation status of KU70 in T-ALL cell lines treated with ERG245, a BCAT1-specific inhibitor [25]. We found a dose-dependent increase in the acetylation status of KU70 (K532) following ERG245 treatment, often associated with pronounced DNA damage (γH2AX) (Figure 4E). Interestingly, the acetylation status of α-tubulin was not significantly altered under these experimental conditions (differently from what was observed using the Class I/II HDAC inhibitor trichostatin A/TSA; Figure 4E). Similarly, mouse *ΔENOTCH1* tumors (which resemble human T-ALL) knockout for *Bcat1* or BCAT1-depleted CCRF-CEM cells showed increased levels of acetylated Ku70/KU70 under basal conditions (Figure 4F and Supplementary Figure S4). KU70 acetylation levels were further increased under DNA-damaging conditions (Supplementary Figure S4) [10]. These results suggest that BCAT1 depletion/inhibition may promote faulty DNA repair through increased acetylation of KU70 (and possibly KU80) proteins.

## 3. Discussion

Although non-canonical functions have previously been assigned to BCAT1 and linked to its redox state [11,16,25], this is the first report to show that BCAT1 utilizes its aminotransferase domain to physically associate with the KU complex of the c-NHEJ pathway and to control DNA repair. Our experiments provide evidence that, following a genotoxic insult by the topoisomerase II inhibitor etoposide (Figure 3C–E), DNA repair pathways c-NHEJ and HR are both activated, but only c-NHEJ is subject to BCAT1 regulation. Depletion of BCAT1 accelerates c-NHEJ as determined by phosphorylation of DNA-PK (Figure 3C), which reaches similar levels as in the presence of BCAT1 but at a faster pace. On the other hand, depletion of BCAT1 leaves HR kinetics unmodified. Ultimately, altering c-NHEJ kinetics increases DNA damage as revealed by a 2–3 fold increase in the levels of γH2AX (Figure 3E), halts the mitotic cycle, and induces apoptosis. The mechanism behind this aberrant DDR in BCAT1-depleted cells is currently unknown; however, we speculate that the consequent accumulation of metabolites (such as 3-HB) and reduced antioxidant buffering capacity may be involved. 

**Figure 5:**
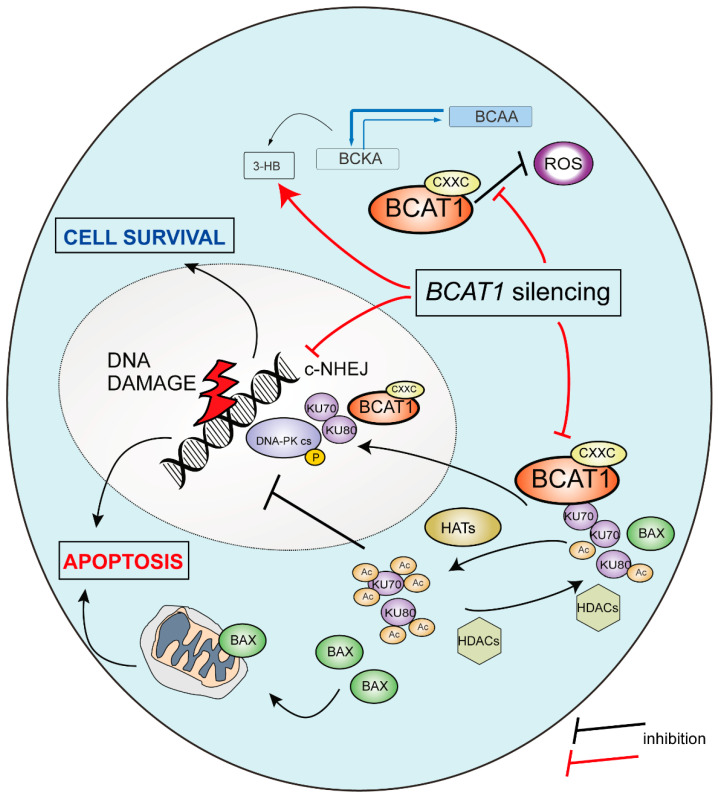
Schematic illustration of a proposed model for BCAT1 in modulating the DNA damage response in T-ALL cells. 3-HB: 3 hydroxy-butyrate; BCAA: branched-chain amino acids; BCKA: branched-chain keto acids; ROS: reactive oxygen species; HATs: histone acetyl transferases; HDACs: histone deacetylases; c-NHEJ: non-homologous end joining. The CXXC motif of BCAT1 implicated in ROS buffering is also shown.

The ability of BCAT1 to complex with KU70 and KU80 suggests that the former directly regulates the c-NHEJ repair pathway. This is an interesting role for a primarily cytosolic enzyme to play on a typically nuclear process. Because our IP experiments utilized ectopic expression of the proteins, it is not immediately clear if such a complex is formed in T-ALL cells, as BCAT1 and the KU70/KU80 proteins are commonly associated with distinct subcellular compartments. To reconcile this paradox, we looked at the subcellular localization of BCAT1 and KU70/KU80 proteins under basal conditions and after a genotoxic insult with etoposide. Our experiments confirm that, under basal conditions, a minor pool of BCAT1 can be found in the nucleus of leukemic cells, whereas KU70 and KU80 proteins are also present in the cytoplasm. This is in line with previous studies, which localized KU70 in the cell membrane and the nucleus of neuroblastoma cells [23] or reported the presence of the KU complex in the cytoplasm of primary CD4+ T cells [26]. Interestingly, cytoplasmic KU70 is known to possess distinct functions from its nuclear counterpart and to bind the pro-apoptotic protein BAX, thus preventing BAX-mediated cell death in neuroblastoma cells [22]. Although colocalization of BCAT1 and KU70/KU80 is possible either in the cytoplasm or the nucleus, PLA assays suggest a weak interaction exists between BCAT1 and KU70 in the cytoplasm of leukemic cells under basal conditions. In contrast, the interaction between BCAT1 and KU70 becomes strong and nuclear following a genotoxic insult. Since depletion of BCAT1 accelerates c-NHEJ, we hypothesize that BCAT1 binds to the KU complex in order to control its redistribution from the cytoplasm to the nucleus and to modulate its nuclear levels and repair activity following DNA damage (Figure 5). Such redistribution of KU70 has previously been reported in neuroblastoma cells after irradiation [22]. The presence of BCAT1 probably limits acetylation of cytoplasmic and nuclear KU70 by restricting the production of 3-HB from leucine [10] (Figure 5). As acetylation of KU70 is associated with induction of apoptosis and poor DNA repair, BCAT1 overexpression in T-ALL is cytoprotective, especially following exposure to DNA-damaging agents. We hypothesized that NOTCH1 directly represses HR [8] and indirectly modulates the rate of c-NHEJ through BCAT1 upregulation in order to promote cell survival in the presence of a genotoxic insult. The same mechanism might be at play during leukemogenesis, which allows for the accumulation of genetic lesions, the survival of the cells, and ultimately the onset of T-ALL. It is worth mentioning here that although our study focused on the function of BCAT1 on DNA repair, we additionally found that BCAT1 was physically associated with numerous ribosomal proteins and might function in RNA splicing. This observation deserves further exploration. In addition, future work needs to be performed to better characterize how the BCAT1-KU70/KU80 interaction regulates DDR in T-ALL cells and the effective function of acetylated KU70 in DNA repair.

Overall, our findings suggest that BCAT1 regulates DNA repair and that targeting BCAT1 could represent a promising therapeutic option, especially in combination with DNA-damaging agents, in T-ALL patients with *NOTCH-1* activating mutations presenting high levels of BCAT1, mostly included in the cortical (CD1a^+^) immunophenotypical subgroup.

## 4. Materials and Methods

### 4.1. Cell Lines and Primary Leukemia Samples

Human embryonic kidney (HEK) 293T cells and U2OS cells were obtained from the American Type Culture Collection (ATCC). These cells were maintained in DMEM containing 10% fetal bovine serum (FBS) and 0.05 mg/mL penicillin/streptomycin. T-ALL cell lines (MOLT-4, Jurkat E6, CCRF-HSB2) were obtained from ATCC, while CCRF-CEM cells were provided by Prof. Adolfo Ferrando (Columbia University, New York, NY, USA). All T-ALL cell lines were maintained in RPMI-1640 media supplemented with 10% FBS and 0.05 mg/mL penicillin/streptomycin. Patient-derived xenografts (PDX) [10] were expanded in vivo via i.v. injection in 6–8 week old female NOD SCID IL2Rγnull (NSG) immunodeficient mice. T-ALL cells from spleens of xenografted mice were cultured in vitro in MEM-alpha media supplemented with 10% human serum and cytokines [27]. *Bcat1* knockout (−/−; KO) mice on a C57BL/6J background were generated using the CRISPR/Cas9 technology by Cyagen (Santa Clara, CA, USA) [10,28]. NOTCH1-induced T-ALL tumors (ΔENOTCH1 tumors) were generated in mice as previously described [29]. Spleens of diseased mice were used as a source of murine T-ALL cells for further studies. Procedures involving animals and their care conformed with institutional guidelines and were authorized by local (OPBA) and national (Italian Ministry of Health) animal ethical committees. 

### 4.2. RNA-Sequencing and Gene-Set Enrichment Analysis

CCRF-CEM shCTRL and CCRF-CEM sh*BCAT1* were treated in triplicate with etoposide (1 μM) or vehicle (DMSO) for 24 h. Next, RNA was extracted using the RNAeasy Mini Kit (#74104; Qiagen, Hilden, Germany), according to the manufacturer’s instructions. Library preparation and paired-end RNA sequencing using Illumina NextSeq 500, as well as downstream data analysis, were performed by Active Motif. Sequenced reads were mapped to the genome using the STAR aligner with default settings, and uniquely mapped reads were counted. Normalized counts per million and differential gene expression were determined with DESeq2.18 (Active Motif). Significantly differentially expressed genes were defined according to a false discovery rate (FDR) and *p* < 0.05 threshold. Hierarchical clustering of Z score and log fold-change expression values used in heatmaps was carried out using GenePattern 2.0 software [30] and iDEP1.1 (http://bioinformatics.sdstate.edu/idep96/ accessed on 9 February 2024). Gene set enrichment analysis (GSEA) was performed on a complete ranked list of our genes using gene sets from the Molecular Signature Database at the Broad Institute (https://www.gsea-msigdb.org/gsea/msigdb/index.jsp accessed on 24 July 2024) [31] using GenePattern software and iDEP1.1. Graphs were plotted using http://www.bioinformatics.com.cn/srplot accessed on 25 July 2024, an online platform for data analysis and visualization. Primary data have been deposited in GEO (GSE275161) and will be released 18 August 2025.

### 4.3. Affinity Purification Mass Spectrometry and Bioinformatics

For the purification of BCAT1-associated proteins, 1 × 10^9^ CUTLL1 cells stably expressing myc-DDK-tagged BCAT1 (BCAT1 myc/DDK) and control CUTLL1 cells were harvested by centrifugation and washed in cold PBS. Cytoplasmic extracts were obtained using hypotonic lysis buffer (Active Motif, Carlsbad, CA, USA) supplemented with phosphatase and protease inhibitor cocktails. The lysates were cleared by centrifugation at 14,000× *g* for 30 min. NaCl concentration in cytoplasmic extracts was adjusted to 150 mM and pre-cleared with Protein G Plus–agarose (sc-2002; Santa Cruz Biotechnology, Dallas, TX, USA) at 4° for 30 min. Approximately 30 mg of cytoplasmic extracts were incubated overnight with EZview Red anti-FLAG M2 affinity gel beads (F2426; Sigma-Aldrich, Merck, Darmstadt, Germany). Beads were washed 3 times with BC100 (20 mM Tris-HCl pH 7.9, 100 mM NaCl, 10% glycerol, 1 mM EDTA) containing 0.3% Triton X-100, and bound proteins were eluted with 4 bead volumes of BC100 containing 0.5 mg/mL of FLAG peptide (F4799; Sigma) for 6 h. The FLAG affinity-purified complexes were further immunopurified by using 30 μL of EZ view Red anti-c-Myc affinity beads (E6654; Sigma). After incubation for 16 h, beads were washed 4 times with BC-100 containing 0.1% Triton X-100 in spin columns [18] and eluted under native conditions using c-Myc peptide (M2435; Sigma). Ten percent of the eluate was resolved on SDS-PAGE and silver-stained using the silverquest kit (LC6070; Invitrogen, Thermo Fisher Scientific, Waltham, MA, USA), while the remaining material was processed for mass spectrometry [27] at the Proteomics Center (University of Padova and Azienda Ospedaliera di Padova). After compiling and filtering the data (proteins representing a fold change (FC) ≥ 10 compared to control, *p*-value ≤ 0.05 in replicates), the protein list was queried on the Kyoto Encyclopedia of Genes and Genomes (KEGG) and hallmark gene sets using ShinyGO software [13] to identify enriched pathways.

### 4.4. Apoptosis Analysis by Flow Cytometry

T-ALL cells (3 × 10^5^) were seeded in 24-well flat-bottom plates and treated with increasing doses of etoposide (100–1000 nM) from Selleck (S1225; Selleck Chemicals LLC., Houston, TX, USA). We analyzed apoptosis after 48–72 h by flow cytometry (FACS) after staining with Annexin V-FITC (APOAF; Roche, Burgess Hill, UK) or Annexin V-PE (#556422; BD Biosciences, Milan, Italy) and SYTOX Red dead cell stain (S34862; Invitrogen). Apoptosis was defined as the sum of the percentage of Annexin V+ and Annexin V+/SYTOX Red+ cells. The samples were collected on a FACSCalibur (BD Biosciences) using Cell Quest software 5.1 (BD Biosciences) or a BD LSR II flow cytometer. Acquired data were analyzed with FlowJo (Tree Star Inc., Ashland, OR, USA). 

### 4.5. Western Blotting and Immunoprecipitation

Total cell lysates were prepared using RIPA lysis buffer supplemented with phosphatase inhibitor cocktail set I and II (P5726, P0044; Sigma-Aldrich, Merck, Darmstadt, Germany) and protease inhibitor cocktail tablets (#05892791001; Roche, Burgess Hill, UK) and normalized for protein concentration using the BCA method (#23227; Pierce, Pero, Italy). For Western blotting, protein samples were separated on 4–12% gradient Tris-Glycine or 3–8% Tris-Acetate SDS-PAGE gels (Invitrogen) and transferred to PVDF membrane (Millipore, Merck, Darmstadt, Germany). Antibodies against tubulin (TU-02; sc-8035), c-myc (9E10; sc-40), and p53 (DO-1; sc-126) were from Santa Cruz Biotechnology (Dallas, TX, USA); antibodies recognizing FLAG epitope (#14793), BCAT1 (#88785), KU-80 (#2180), KU-70 (#4588), phosphorylated H2AX (pS139; #9718), phosphorylated DNA-PKcs (pS2056; #68716), total DNA-PKcs (#12311), phosphorylated ATM (pS1981; #5883), total ATM (#2873), phosphorylated CHK1 (pS345; #2348), phosphorylated CHK2 (pT68; #2197), total CHK2 (#6334), phosphorylated TP53 (pS15; #12571), and GADPH (#5174) were from Cell Signaling Technology (Danvers, MA, USA). Mouse anti-BCAT1 (#611271; BD Pharmingen, Oxford, UK) and rabbit anti-Green Fluorescent Protein (GFP) antibody (#A-11122) were from Invitrogen. Rabbit polyclonal antibody against acetyl-KU70 (Lys542; #OASG04124) was from Aviva Systems Biology (San Diego, CA, USA). The BioRad ChemiDoc XRS Imager was used to capture the signals from the blots. We quantified each protein band using ImageJ 1.54f software and normalized each target protein (after background subtraction) to its loading control or to its total protein form (for phosphorylated proteins). For co-immunoprecipitation (CO-IP) assays, HEK 293T cells stably expressing BCAT1 myc/DDK were transfected with expression vectors encoding pEGFP-C1-FLAG-Ku70 (Addgene #46957) or pEGFP-C1-FLAG-Ku80 (Addgene #46958) using JetPEI transfection reagent (#101000020; Polypus-transfection Inc., New York, NY, USA). Forty hours after transfection, cells were lysed in NETN lysis buffer (10 mM Tris-HCl pH 7.5, 150 mM NaCl, 0.5 mM EDTA, 0.5% NP-40, and protease inhibitor cocktail). For IP, cell lysates were pre-cleared with Protein G–agarose (Santa Cruz Biotechnology) at 4 °C for 30 min before being incubated with GFP-Trap agarose (#gta-20; Proteintech, Manchester, UK), or EZ view Red anti-c-Myc affinity beads (Sigma-Aldrich) overnight at 4 °C. Beads were washed five times with lysis buffer and boiled in 2×SDS Laemmli’s sample buffer. Immune complexes were analyzed by SDS-PAGE and immunoblot. For experiments mapping the regions of interaction between BCAT1 and XRCC5 or XRCC6, HEK 293T cells stably expressing BCAT1 myc/DDK were transfected with expression vectors for XRCC6 (pcDNA-HAXRCC6 (full-length: 1–609), N-terminal (1–350), Ku domain (351–500), C-terminal (501–609)) or XRCC5 (pLenti-HAXRCC5 (full-length: 1–732), N-terminal (1–240), Ku domain (241–550), C-terminal (551–732)). Forty hours after transfection, cells were lysed in modified NETN lysis buffer (10 mM Tris-HCl pH 7.5, 150 mM NaCl, 0.5 mM EDTA, 1% Triton-X, and protease inhibitor cocktail). Before IP, cell lysates were pre-cleared with Protein G–agarose (Santa Cruz Biotechnology) at 4 °C for 30 min before being incubated with EZ view Red anti-FLAG affinity beads (Sigma-Aldrich) overnight at 4 °C. Beads were washed five times with lysis buffer and boiled in 2×SDS Laemmli’s sample buffer. Immune complexes were analyzed by SDS-PAGE and immunoblot. For experiments mapping the regions of interaction between XRCC5 or XRCC6 and BCAT1, HEK 293T cells were co-transfected with expression vectors for full-length XRCC6 (pcDNA-HAXRCC6 (1–609)) or full-length XRCC5 (pLenti-HAXRCC6 (1–732)) and BCAT1 deletion mutants in the pLenti-Myc-DDK-P2A-Puro vector (N-terminal (1–197), C-terminal (198–373), AT-IV domain (94–343)). Forty hours after transfection, cells were lysed in modified NETN lysis buffer. Before IP, cell lysates were pre-cleared with Protein G–agarose (Santa Cruz Biotechnology) at 4 °C for 30 min before being incubated with EZ view Red anti-HA affinity beads (E6779; Millipore Sigma-Aldrich) overnight at 4 °C. Beads were washed five times with lysis buffer and boiled in 2× SDS Laemmli’s sample buffer. Immune complexes were analyzed by SDS-PAGE and immunoblot. For IP of endogenous BCAT1-bound proteins in T-ALL cells, we lysed 100–150 × 10^6^ CUTLL1 T-ALL cells engineered to express myc/DDK-tagged BCAT1 (CUTLL1 BCAT1-myc/DDK) for 30 min in RIPA lysis buffer supplemented with phosphatase and protease inhibitor cocktails. After centrifugation, cell lysates (diluted 1:3 with modified NETN buffer) were pre-cleared with Protein G–agarose (Santa Cruz Biotechnology) at 4 °C for 30 min before being incubated overnight with anti-FLAG M2 affinity gel beads (Sigma-Aldrich). Beads were washed three times with modified NETN lysis buffer, and proteins were eluted by incubating the beads with FLAG peptide (1 mg/mL, Sigma-Aldrich). Further, for detecting endogenous interactions, CCRF-CEM cells were lysed in NETN lysis buffer. After centrifugation, the cell lysates were pre-cleared with TrueBlot^®^ anti-Mouse Ig IP Beads (#00-8811-25; Rockland Immunochemicals Inc., Limerick, PA, USA) before being incubated overnight with 5 µg of mouse antibody against BCAT1 (ECA39, sc-517185; Santa Cruz Biotechnology) or irrelevant mouse Ig (sc-2025; Santa Cruz Biotechnology). Subsequently, samples were incubated for 2 h with TrueBlot^®^ anti-Mouse Ig IP Beads, and IP washed 5 times with NETN lysis buffer. Immune complexes were then analyzed by SDS-PAGE and immunoblot.

### 4.6. Plasmids, Lentiviral Constructs, and Viral Production

For *BCAT1* silencing experiments, HEK293T cells were transfected with pGipz non-silencing shRNA control (RHS4346), sh*BCAT1*#1 (RHS4430-200295245), sh*BCAT1*#2 (RHS4430-200207676), pLKO.1-sh*BCAT1* (#3; TRCN0000005907; Sigma-Aldrich), pLKO.1-puro shcontrol (SHC002; Sigma-Aldrich), and appropriate packaging plasmids using JetPEI transfection reagent (Polyplus, Illkirch, France). BCAT1 overexpression was performed using pLenti-BCAT1-Myc-DDK-P2A-Puro (RC219229L3; Origene, Rockville, MD, USA). Plasmids carrying deletion mutants of BCAT1 (N-terminal, C-terminal, and AT-IV domain) were synthesized and cloned in the pLenti-Myc-DDK-P2A-Puro vector using AsiSI and MluI restriction sites by Genewiz (ALENTA Life Sciences, Chelmsford, MA, USA). Plasmids encoding HA-tagged XRCC5 full-length and deletion mutants (N-terminal, C-terminal, and Ku domain) were synthesized and cloned in the pLenti-P2A-Puro vector using AsiSI and MluI restriction sites by Genewiz (ALENTA Life Sciences, Chelmsford, MA, USA). pcDNA-HA-XRCC6 full-length and deletion mutants (N-terminal, C-terminal, and Ku domain) were kindly donated by Bae Jeehyon (Seoul, Korea) [32]. For viral production, viral supernatant from transfected cells was collected 48 h after transfection, filtered, and used to infect target cells. All infections of T-ALL cells were performed by spinoculation. After infection, T-ALL cells were selected for 3–7 days in puromycin before functional assays.

### 4.7. Analysis of Double Strand Breaks (DSB) Repair by Reporter Assays

The efficacy of DNA double-strand repair was determined using GFP-based reporter assays as described previously [18,19]. Briefly, cells (U2OS, Jurkat E6) were transfected with pimEJ5-GFP (Addgene #44026) vectors. The transfected cells were selected in puromycin (1 mg/mL) for at least two weeks. Selected T-ALL reporter cell lines were infected with lentiviral particles for pLKO.1-sh*BCAT1* or pLKO.1-puro shcontrol. U2OS reporter cell lines were instead infected with lentiviral particles for pLenti-Myc-DDK-P2A-Puro empty vector, pLenti-BCAT1-Myc-DDK-P2A-Puro, or BCAT1 mutants (catalytically dead mutant where lysine 222 is mutated to alanine, i.e., BCAT1K222A, or redox motif inactive mutant BCAT1SXXS where cysteines 335 and 338 in the CXXC motif are mutated to serines). Five to ten days post-infection, cell lines were evaluated for gene editing, i.e., *BCAT1* silencing or overexpression. Cell lines with successful modulation of BCAT1 expression were transfected/electroporated with the SceI endonuclease expression vector (pCBA-SceI; Addgene #26477) to induce DSBs. Forty-eight to seventy-two hours after the induction of DSBs, cells were analyzed by flow cytometry FACSCalibur (BD Biosciences). The percentage of GFP-positive cells was used as an indication of c-NHEJ efficiency.

### 4.8. Cellular Fractionation

For cell fractionation analysis, nuclear and cytoplasmic fractions were collected using the Nuclear Extract Kit (#40010; Active Motif, Carlsbad, CA, USA) following manufacturer instructions.

### 4.9. Immunoprecipitation of Acetylated Proteins

Immunoprecipitation of acetylated proteins was performed using the Signal-seeker Acetyl-Lysine detection kit (SKU BK163; Cytoskeleton, Inc., Denver, CO, USA) according to the manufacturer’s recommendations. Briefly, cells were lysed in diluted BlastR lysis buffer containing class I and II HDAC inhibitors (trichostatin A; TSA) and class III HDAC inhibitors (nicotinamide). Approximately 2 mg of protein was pre-cleared with Protein G–agarose (Santa Cruz Biotechnology) at 4 °C for 30 min before being incubated with acetyl-lysine affinity beads or acetyl-lysine IP control beads overnight at 4 °C. Beads were washed three times with BlastR-2 wash buffer, and bound proteins were eluted with bead elution buffer. Immune complexes were analyzed by SDS-PAGE and immunoblot.

### 4.10. Immunofluorescence Analysis

T-ALL cells were seeded on Shi-fix coverslips (SB-Shifix50PLUS; Shikhar Biotech, Nepal) according to the manufacturer’s instructions. After washing in PBS, cells were fixed with 4% paraformaldehyde and permeabilized with PBS-0.1% Triton-X (Sigma-Aldrich). Cells were blocked in PBS-1% goat serum (S-1000-20; Vector Laboratories, Newark, NJ, USA) and incubated with rabbit antibodies against KU70 (10723-1-AP; 1:80; Proteintech Europe) and a mouse antibody against BCAT1 (1:80; BD Pharmingen) overnight at 4 °C. Subsequently, samples were washed and incubated with secondary antibodies: anti-rabbit Alexa Fluor 568 (A-11011; 1:500; Invitrogen) and anti-mouse Alexa Fluor 488 (A-11001; 1:500; Invitrogen) at room temperature for 1 h in the dark. After washing with PBS, DAPI staining was performed to visualize nuclear DNA. Images were acquired with a Zeiss LSM900 confocal microscope equipped with argon (488 nm) and helium-neon (543 nm and 633 nm) laser sources and a 63X PlanFluo oil immersion objective.

### 4.11. Analysis of 53BP1 and γH2AX Foci by Immunofluorescence

T-ALL cells were seeded on Shi-fix coverslips (Shikhar Biotech, Nepal) according to the manufacturer’s instructions. Staining was performed as previously described [9]. After washing in PBS, cells were fixed with 4% paraformaldehyde and permeabilized with PBS-0.1% Triton-X (Sigma-Aldrich). Cells were blocked in PBS-1% goat serum (Vector Laboratories, Newark, NJ, USA) and incubated with rabbit antibodies against 53BP1 (NB100-304; 1:500; Novus Biological, Bio-Techne SRL, Milano, Italy) and a mouse antibody against γH2AX (05-636-I; 1:250; Millipore) overnight at 4°C. Subsequently, samples were washed and incubated with secondary antibodies: anti-rabbit Alexa Fluor 647 (A-21244; 1:500; Invitrogen) and anti-mouse Alexa Fluor 568 (A-11004; 1:500; Invitrogen) at room temperature for 1 h in the dark. After washing with PBS, DAPI staining was performed to visualize nuclear DNA. Images were acquired with a Zeiss LSM900 confocal microscope equipped with argon (488 nm) and helium-neon (543 nm and 633 nm) laser sources and a 63X PlanFluo oil immersion objective. Foci were counted using ImageJ software.

### 4.12. Proximity Ligation Assay (PLA)

T-ALL cells untreated or treated with etoposide (1 µM) for 24 h were seeded on Shi-fix coverslips (Shikhar Biotech), washed with PBS, and then fixed and permeabilized as above. Cells were then subjected to in situ PLA using Duolink Detection Reagents Red (DUO92008, Sigma-Aldrich) according to the manufacturer’s instructions. Primary antibodies used were rabbit antibodies against KU70 (1:50; Proteintech Europe, Manchester, UK) and a mouse antibody against BCAT1 (1:50; BD Pharmingen, Oxford, UK) overnight at 4 °C. Duolink anti-mouse PLA probe MINUS (DUO92004, Sigma-Aldrich) and Duolink anti-rabbit PLA probe PLUS (DUO92002, Sigma-Aldrich) were used. DAPI staining was performed to visualize nuclear DNA.

### 4.13. Statistical Analyses

Results were expressed as mean value ± standard deviation (SD). Normality checks of analyzed data were performed using the Shapiro–Wilk test. For analyses comparing multiple experimental groups, the Kruskal–Wallis test (non-parametric test) was used. Student’s *t*-test and the nonparametric Mann–Whitney *U* test were used where appropriate. We calculated half inhibitory concentrations (IC50) with CompuSyn 1.0 software (Biosoft, Cambridge, UK) [33]. All statistical tests were two-sided, unpaired, and *p* < 0.05 was considered statistically significant (* *p* < 0.05, ** *p* < 0.01, *** *p* < 0.001). Statistical analyses were performed with GraphPad Prism 8.0 software (GraphPad Software, San Diego, CA, USA).

## Data Availability

The data presented in this study are available on request from the corresponding author.

## Supplementary Materials

The following supporting information can be downloaded at: https://www.mdpi.com/article/10.3390/ijms252413571/s1.
