# Supplementary material for: BCAT1 Associates with DNA Repair Proteins KU70 and KU80 and Contributes to Regulate DNA Repair in T-Cell Acute Lymphoblastic Leukemia (T-ALL)
Source: Int J Mol Sci. 2024 Dec 18;25(24):13571. doi: 10.3390/ijms252413571 (PMC11676169; doi:10.3390/ijms252413571)
Supplement: Supplementary file 1 [file ijms-25-13571-s001.zip › Supplementary Information_DNAdamage_FINAL_REV_OK.pdf]

International Journal of
*Molecular Sciences*

MDPI

*Supplementary Information*

# BCAT1 associates with DNA repair proteins KU70 and KU80 and contributes to regulate DNA repair in T-cell acute lymphoblastic leukemia (T-ALL)

**Valeria Tosello, Chiara Rompietti, Adonia Papathanassiu, Giorgio Arrigoni and Erich Piovan**

**Supplementary Information**

**-Supplementary Table S1:** Differentially expressed genes between CCRF-CEM shBCAT1 and control cells
**-Supplementary Table S2:** Differentially expressed genes between CCRF-CEM shBCAT1 treated with etoposide and control cells treated with etoposide
**-Supplementary Table S3**: BCAT1 interacting partners identified by Affinity purification mass spectrometry
**-Supplementary Figure S1.** BCAT1 loss sensitizes cells to apoptosis following etoposide treatment
**-Supplementary Figure S2.** Cellular distribution of BCAT1 and KU proteins in T-ALL cells
**-Supplementary Figure S3.** BCAT1 interacts with KU80 protein
**-Supplementary Figure S4**. BCAT1 depletion increases KU-70 acetylation

A

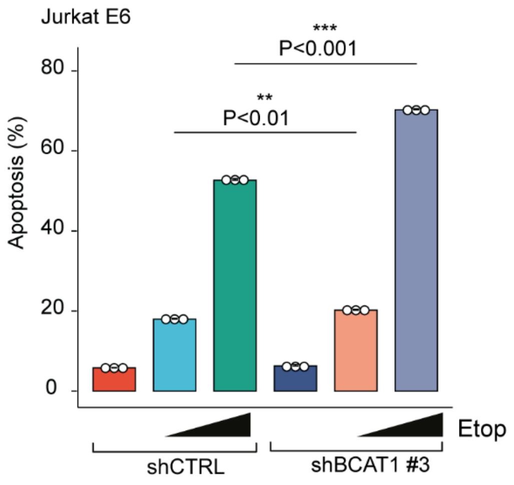


B

| | | IC$_{50}$ (nM) | SD |
|---|---|---|---|
| **CEM** | shCTRL | 3028.06 | 849.8292 |
| | shBCAT1 #1 | 359.057 | 40.01659 |
| | shBCAT1 #2 | 405.2155 | 26.97683 |
| **Jurkat** | shCTRL | 1446.52 | ND |
| | shBCAT1 #3 | 617.73 | ND |

**Supplementary Figure S1.** BCAT1 loss sensitizes cells to apoptosis following etoposide treatment. (**A**) Quantification of apoptosis in Jurkat E6 T-ALL cells transduced with shCTRL or sh*BCAT1* (#3) and treated in vitro for 48h with DMSO (vehicle) or Etoposide (250 nM– 1 µM). Significance was calculated using an unpaired two-tailed t-test. ** $p< 0.01$, ***$p< 0.001$. (**B**) Half inhibitory concentration (IC$_{50}$) for Etoposide in CCRF-CEM and Jurkat T-ALL cell lines transduced with shCTRL or sh*BCAT1* targeting vectors. SD:Standard deviation of IC$_{50}$ values obtained from two independent experiments. ND: Not determined.

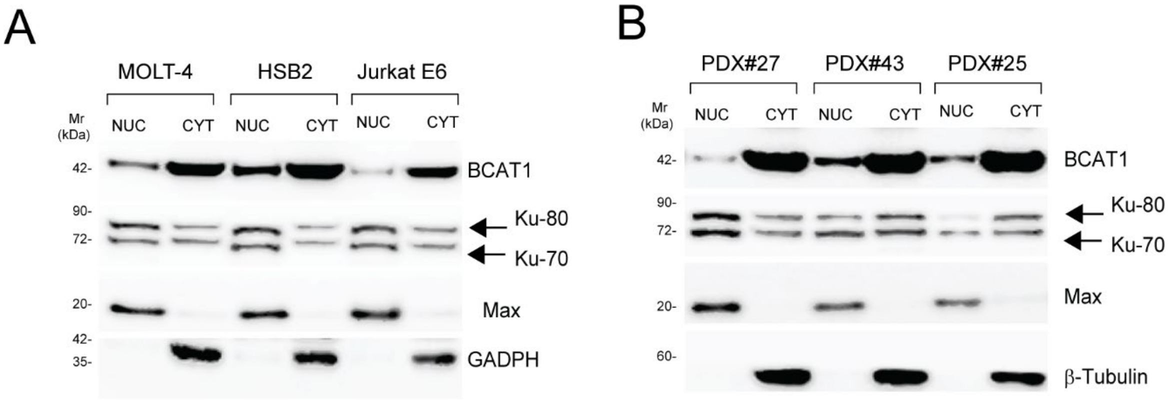


**Supplementary Figure S2.** Cellular distribution of BCAT1 and KU proteins in T-ALL cells. (**A**) Cellular localization analysis of BCAT1, KU70, KU80 via Western blot analysis of nuclear and cytoplasmic cell fractions in cell lysates from MOLT-4, CCRF-HSB2, and Jurkat E6 T-ALL cells. GADPH and Max proteins are shown as controls for cytosolic and nuclear fractions. CYT: cytoplasmic fraction; NUC: nuclear fraction. (**B**) Cellular localization analysis of BCAT1, KU70, KU80 via Western blot analysis of nuclear and cytoplasmic cell fractions in cell lysates from patient derived xenograft cells (PDX; PDX#27, PDX#43, PDX#25). Tubulin and Max proteins are shown as controls for cytosolic and nuclear fractions. CYT: cytoplasmic fraction; NUC: nuclear fraction.

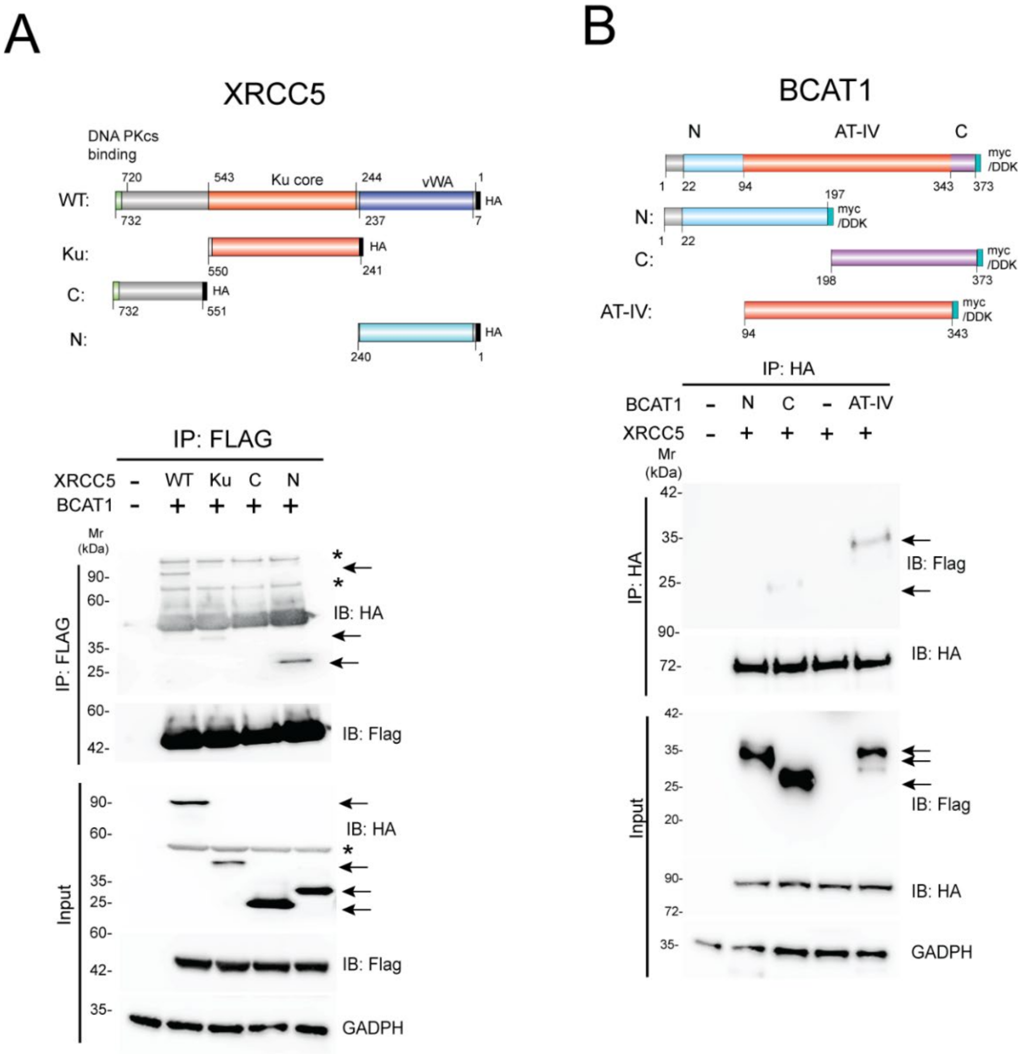


**Supplementary Figure S3.** BCAT1 interacts with KU80 protein. (**A**) Schematic representations of the plasmids encoding full-length (WT) and truncation mutants of XRCC5 (top). vWA: von Willebrand A domain. HEK 293T cells stably expressing epitope-tagged BCAT1 were transfected with the indicated plasmid. Cell lysates were subjected to IP with anti-FLAG beads followed by immunoblot analysis with the indicated antibodies. (**B**) Schematic representations of the plasmids encoding full-length (WT) and truncation mutants of BCAT1 (top). N: Branched chain amino acid aminotransferase-like N-terminal domain; AT-IV: aminotransferase class IV domain; C: Branched chain amino acid aminotransferase-like C-terminal domain. HEK 293T cells were transfected with HA-tagged XRCC5 and the indicated BCAT1 mutant plasmids. Cell lysates were subjected to IP with anti-HA beads followed by immunoblot analysis with the indicated antibodies. The arrows indicate expected positions of the respective proteins, and asterisks (*) indicate non-specific bands.

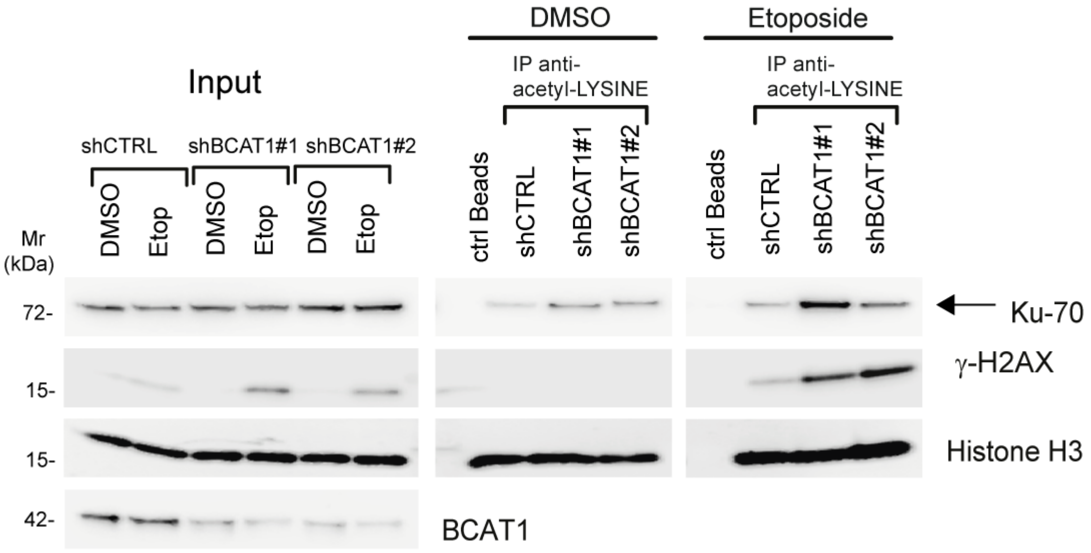


**Supplementary Figure S4.** BCAT1 depletion increases KU-70 acetylation. CCRF-CEM T-ALL cells transduced with shCTRL or shBCAT1 (#1 and #2) were treated with 1 μM etoposide for 24h, subsequently whole cell lysates were collected and immunoprecipitated using anti-acetyl-lysine affinity beads or control beads and probed for KU70, γH2AX. Total H3 was used as loading control (for input).
